# Digital Migration of the Loewenstein Acevedo Scales for Semantic Interference and Learning (LASSI-L): Development and Validation Study in Older Participants

**DOI:** 10.2196/64716

**Published:** 2025-02-19

**Authors:** Philip Harvey, Rosie Curiel-Cid, Peter Kallestrup, Annalee Mueller, Andrea Rivera-Molina, Sara Czaja, Elizabeth Crocco, David Loewenstein

**Affiliations:** 1 Psychiatry and Behavioral Sciences, University of Miami Miller School of Medicine, 1120 NW 14th Street, Miami, FL, 33136, United States, 1 3052434094; 2 i-Function, Inc, Miami, FL, United States; 3 Geriatrics and Palliative Medicine, Weill Cornell Medicine, New York, NY, United States

**Keywords:** mild cognitive impairment, cognitive challenge tests, elder, aging, amyloid biomarkers, cognition, cognitive decline, deterioration, semantic interference, Alzheimer disease, self-administered, voice recognition, technology, assessment study, accuracy, artificial intelligence, treatment, medication, mental health, biomarkers, amnesia, neurodegeneration, patient health, health monitoring, digital mental health, neuroscience, neurotechnology, Loewenstein Acevedo Scales for Semantic Interference and Learning, LASSI-L, digital Loewenstein-Acevedo Scales for Semantic Interference, LASSI-D

## Abstract

**Background:**

The early detection of mild cognitive impairment is crucial for providing treatment before further decline. Cognitive challenge tests such as the Loewenstein-Acevedo Scales for Semantic Interference and Learning (LASSI-L) can identify individuals at highest risk for cognitive deterioration. Performance on elements of the LASSI-L, particularly proactive interference, correlate with the presence of critical Alzheimer disease biomarkers. However, in-person paper tests require skilled testers and are not practical in many community settings or for large-scale screening in prevention.

**Objective:**

This study reports on the development and initial validation of a self-administered computerized version of the Loewenstein-Acevedo Scales for Semantic Interference (LASSI), the digital LASSI (LASSI-D). A self-administered digital version, with an artificial intelligence–generated avatar assistant, was the migrated assessment.

**Methods:**

Cloud-based software was developed, using voice recognition technology, for English and Spanish versions of the LASSI-D. Participants were assessed with either the LASSI-L or LASSI-D first, in a sequential assessment study. Participants with amnestic mild cognitive impairment (aMCI; n=54) or normal cognition (NC; n=58) were also tested with traditional measures such as the Alzheimer Disease Assessment Scale-Cognition. We examined group differences in performance across the legacy and digital versions of the LASSI, as well as correlations between LASSI performance and other measures across the versions.

**Results:**

Differences on recall and intrusion variables between aMCI and NC samples on both versions were all statistically significant (all *P*<.001), with at least medium effect sizes (*d*>0.68). There were no statistically significant performance differences in these variables between legacy and digital administration in either sample (all *P*<.13). There were no language differences in any variables (*P*>.10), and correlations between LASSI variables and other cognitive variables were statistically significant (all *P*<.01). The most predictive legacy variables, proactive interference and failure to recover from proactive interference, were identical across legacy and migrated versions within groups and were identical to results of previous studies with the legacy LASSI-L. Classification accuracy was 88% for NC and 78% for aMCI participants.

**Conclusions:**

The results for the digital migration of the LASSI-D were highly convergent with the legacy LASSI-L. Across all indices of similarity, including sensitivity, criterion validity, classification accuracy, and performance, the versions converged across languages. Future studies will present additional validation data, including correlations with blood-based Alzheimer disease biomarkers and alternative forms. The current data provide convincing evidence of the use of a fully self-administered digitally migrated cognitive challenge test.

## Introduction

Diagnosis of common cognitive neurodegenerative illnesses such as Alzheimer disease (AD) and Alzheimer disease–related disorders (ADRD) has typically relied on clinical impressions and traditional cognitive assessments. Given the rising prevalence of these diseases, and the continuing development of disease modifying therapies, it is important to detect the earliest cognitive manifestations of disease before multisystem damage has occurred. Traditional neuropsychological measures, such as those involving memory, are based on paradigms over 5 decades old and may not be sensitive to the earliest cognitive changes that accompany AD/ADRD [1-3]. Early detection of cognitive deficits in AD/ADRD provides the best opportunities for interventions, as current and emerging therapies are likely to be considerably more efﬁcacious before the onset of multisystem pathology. Thus, a recent focus has been on the development of “cognitive stress” [4,5] or “cognitive challenge” tests that tax compensatory mechanisms that may reveal the earliest clinical changes associated with AD/ADRD.

One such measure is the extensively studied Loewenstein-Acevedo Scales of Semantic Interference and Learning (LASSI-L) [6]. This paradigm taps vulnerability to proactive semantic interference, which is known to increase in older individuals [7]. The first task in the LASSI-L is for participants to learn a list of 15 words belonging to 3 semantic categories, (list A). A competing semantically similar set of targets (list B) is then presented. As a result, proactive semantic interference (PSI) can be measured, which is a type of interference that occurs when previously learned information interferes with the ability to learn and remember new, semantically related, information. These effects are indexed by reduced learning of the words in list B and possible intrusions of list A words into the recall of list B. A unique feature of the LASSI-L is a second presentation of the second target list that taps failure to recover from proactive semantic interference (frPSI) [8], indexed by continued learning deficits after a second presentation of the B targets and continued increases in the relative number of intrusions from list A.

The LASSI-L frPSI measure has been found to be highly related to total and regional amyloid load in apparently neuropsychologically normal community-dwelling elders [9,10]; to differentiate between aMCI patients with suspected AD from cognitively unimpaired elderly controls (cognitively normal) [6,11,12]; and has been associated with volumetric loss in AD-prone areas among elders with aMCI [13,14]. In contrast, other LASSI-L indices and standard memory tests were not related to volumetric ﬁndings and progression to mild cognitive impairment (MCI) [15,16]. Particularly relevant to AD/ADRD, are findings that semantic intrusion errors on the PSI and frPSI indices of the LASSI-L are greater in persons with aMCI who are amyloid positive on positron emission tomography compared with individuals with aMCI due to other neurological or neuropsychiatric conditions, who are amyloid negative [17,18]. This indicates a remarkable ability of the LASSI-L to differentiate those with AD pathology from those without. The LASSI-L has also differentiated between persons who are amyloid positive versus amyloid negative on the basis of other AD biomarkers such as plasma p-tau 181 [19] as well as plasma AB42/AB40 ratios using mass spectrometry.

A critical pragmatic factor associated with the LASSI-L is its cross-cultural sensitivity. Spanish and English versions of the test are equivalently valid [20-22] and have shown both multilingual and cross-cultural validity. For example, in a study in Madrid, Spain, performance on the LASSI-L was associated with regional cerebral metabolic deficits associated with AD on positron emission tomography [23]. Furthermore, the LASSI-L has shown specificity to aMCI in African American populations [24], suggesting that it has wide ranging cross-cultural applicability. In the Rosselli et al [22] study, it was found that bilingual Spanish-English speakers outperformed monolingual Spanish speakers, suggesting that testing language is an important consideration when persons are bilingual.

Although the LASSI-L is both relatively brief and administered with high reliability by examiners, its deployments for large-scale screening, such as for clinical trials, particularly those focused on prevention, may be limited by inadequate professional resources to administer the test. The initial research-focused computerized version of the LASSI-L, the Loewenstein-Acevedo Scales for Semantic Interference and Learning, Brief Computerized Version is both abbreviated and computerized, eliminating delayed recall conditions and enhancing validity of test administration. The initial studies of this assessment found that it had excellent convergence with the LASSI-L and sensitivity to aMCI and pre-MCI [25,26], as well as sensitivity to AD biomarkers during prodromal stages of the disease [27]. However, the Loewenstein-Acevedo Scales for Semantic Interference and Learning, Brief Computerized Version still requires a trained psychometrist to supervise the assessment, and manual scoring to ensure that the data captured by computer match the data captured by the human.

In this paper, we report on the further digital migration of the LASSI-L through development of a fully self-administered, computerized version of the Loewenstein-Acevedo Scales for Semantic Interference (LASSI), the LASSI-D. Using cloud-based administration and an AI-generated humanoid avatar testing assistant, the LASSI-D was examined for its convergent and discriminative validity in a structured digital migration study. The migration of the task from in person and paper to computerized self-administration is a critical first step in demonstrating convergent validity of the paper-and-pencil LASSI-L with the computerized LASSI-D. As part of the development process, we developed an identical digital version of the LASSI-L, with identical stimuli (designated LASSI-D, form A). An exploratory additional feature of the development process was to create 2 alternative forms by using the same stimuli, but reorganizing their order of appearance across target lists.

We administered the LASSI-L or the LASSI-D first in a sequential design, to community-dwelling individuals, who first received an in-person cognitive assessment for categorization of their cognitive status. In addition, at the LASSI assessment for both cohorts, we administered several standard cognitive measures used for staging AD/ADRD, including immediate and delayed paragraph recall [28], the Hopkins Verbal Learning Test-revised [29], and Alzheimer Disease Assessment Scale-Cognition (ADAS-Cog) [30].

Our overall study goals were to examine convergence of the LASSI-D form A with standard administration, and the LASSI-L, in terms of performance. Further we assessed validity in terms of the convergence of the Spanish and English versions of each test, and to explore the correlations of the LASSI-L and the LASSI-D with more traditional assessments of cognition related to aMCI.

## Methods

### Overall Study Design

This was the first part of a longitudinal observational study conducted at 7 community centers or locations in South Florida and 4 in New York City. Following screening and orientation to the study, participants received an in-person assessment to characterize their cognitive status. Participants who met inclusion criteria and adequately completed the initial assessment progressed to later parts of the study.

### Participants

The sample included English- or Spanish-speaking adults aged 59 years or older who lived in the community, had at least 20/60 vision, were able to read a computer screen, and had adequate hearing for an auditory-based assessment. Men and women were recruited, without restrictions on racial or ethnic status. MCI status was ascertained with a neuropsychological assessment using the Jak-Bondi criteria [31].

Exclusion criteria included a Montreal Cognitive Assessment (MoCA) [32] score of <19 or a reading score, in their dominant language, at less than a 6th grade level. Other exclusions included the inability to undergo assessments in either English or Spanish, having been tested with the LASSI-L in the past 12 months, a diagnosis of a serious psychiatric condition apart from clinical depression, a previous medical history of brain disorder such as stroke, seizures, tumor, or significant traumatic brain injury with extended loss of consciousness.

### Reading Performance

The literacy level of English speakers was examined with the Wide Range Achievement Test [33], third edition. Spanish speakers were assessed with the Batería III Woodcock-Munoz Identificacion de letras y palabras subtest [34].

### Cognitive Assessments

Cognitive assessments were used to collect data for the performance-based MCI criteria. The LASSI measures were not used in diagnostic determination. All assessments were performed in each participants’ preferred language (English or Spanish). Montreal Cognitive Assessment (MoCA [32]) examines cognitive performance with scores ranging from 0‐30. Assessments were performed by certified bilingual raters. Using Wechsler Memory Scale- revised, Logical Memory I and II (Anna Thompson Story) [28], participants were asked to read a story and then asked for immediate recall, followed by a 20-minute delayed recall filled with other nonverbal assessments. Hopkins Verbal Learning Test-Revised [29] was administered before assessment with the Brief Assessment of Cognition (BAC), substituting this shorter 12-item, 3 trial verbal learning assessment for the much harder 15-item 5-trial BAC word list test. We also examined delayed recall to contribute to the MCI classification.

Wechsler Memory Scale- revised, Logical Memory I and II (Anna Thompson story) [28]. Participants were asked to read a story and then asked for immediate recall, followed by a 20 -minute delayed recall filled with other non-verbal assessments.

The BAC app version [35] measures domains of cognition known to be related to everyday functioning. The BAC app delivers the same assessments with cloud-connected tablet delivery for ease of administration and standardization. The BAC-app has been widely used in studies of MCI. The cognitive domains assessed include: Digit Sequencing, Token Motor; Verbal Fluency; Symbol Coding; Tower of London. Participants with MoCA scores less than 19 were not tested further and testing also stopped if participants did not have the required literacy level.

### Warm-Up Testing Before LASSI Assessments

Two short tests, Trail-Making Test part A and Animal Naming Fluency were administered immediately before the LASSI-L and the LASSI-D. This assessment was designed to ensure that the participants were ready to be assessed with the somewhat longer LASSI procedure and to provide additional information about the similarities of the participant samples. Performance on these tests were not used for participant selection.

### The LASSI

Participants were examined with 2 variants of the LASSI: the legacy technician delivered paper version: the LASSI-L and the LASSI-D, which has identical stimuli to the LASSI-L. The LASSI uses controlled learning and cued recall to maximize encoding of a list of to-be-remembered target words representing three semantic categories. The examinee learns a list of 15 common words comprised of musical instruments, fruits, or articles of clothing (5 words per semantic category) which they are asked to recall immediately after exposure. Critically, exposure to stimuli occurs with the participant being shown each of the words, one at a time, and being asked to read the word aloud, thus ensuring encoding of every stimulus. Words that were read incorrectly are immediately cued in the following way: “The word is XYZ. Please say XYZ.”

After the free recall trial, the examinee is presented with each category cue (eg, clothing) and asked to recall the words from that category, yielding a cued recall score (cued A1). The examinee is then exposed to the same target stimuli for a second learning trial with subsequent cued recall, with a goal of strengthening the acquisition and recall of the list A targets, providing maximum encoding of the to-be-remembered information (cued A2). As the LASSI-L targets identification of semantic intrusions in individuals without major memory challenges, previous studies of the LASSI-L have excluded participants who were unable to learn at least 11 of the 15 list A stimulus words [18-20].

**Figure 1.**
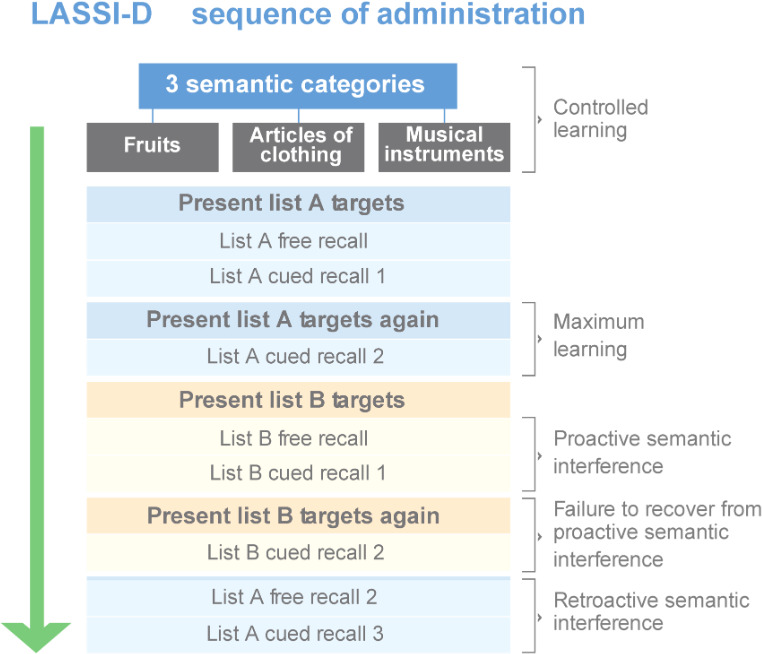
Assessment flow within Loewenstein-Acevedo Scales for Semantic Interference Assessments. LASSI-D: digital Loewenstein-Acevedo Scales for Semantic Interference.

Following the second presentation of list A, the participant is presented a semantically related list (list B) which consists of 15 words which are different from list A, with 5 from each of the same categories and again asked to read them aloud. Following the list B presentation, the examiner asks the participant for free recall of the list B words (B1 free recall); reduced learning compared with list A defines immediate proactive semantic interference (PSI). Then, the participants are cued to recall the list B words. Next, the participants read the list B words again, followed by a second cued recall trial (cued recall B2). Recall performance on this second learning trial measures whether the examinee’s manifests failure to recover from PSI (frPSI). Refer to Figure 1 for the sequence of assessments contained in the LASSI.

An additional critical set of outcomes are intrusion scores. During both free and cued recall conditions for list B any recalled words that are not from the are recorded. This dependent variable is calculated into an index, dividing the number of intrusion errors by the total words recalled, including words correctly recalled and intrusions, yielding “percentage of intrusion errors” (PIE) This index captures the proportion of all recalled information that constitutes intrusions. PIE has been shown to be the most sensitive predictor of risk for subsequent cognitive decline [16,36]. The LASSI-L has been translated into multiple languages, including Spanish. We used the English and Spanish versions in this study.

### Digital Migration of the LASSI-L

The digital assessments with the LASSI-D were computerized, cloud-based, and completely self-administered. To ensure that the LASSI-D is a valid equivalent of the LASSI-L, several important steps were undertaken in the migration. The first was piloting and refining the (Google) voice recognition software through pilot testing to ensure accurate of identification of words read and recalled by participants (taking into consideration different English and Spanish accents, physical environments, etc). Second was the development of a realistic AI generated humanoid avatar. The avatar vocalizes the instructions and feedback during assessment that are read by the human tester during LASSI-L assessments. Third was the development of a digitally administered pretesting orientation and preparation session to ensure attentiveness and understanding on the part of the participant. This orientation serves to ready the user to properly interface with the LASSI-D application, while testing the functionality of the user’s internet connection, the microphone, and speakers. In the orientation, the participant is asked to name some simple shapes that appear on the device’s screen, continuing until correct responses are detected by the software. Thus, the resulting software program interactively provides instructions and feedback to participants, presenting all stimuli and identifying and collating the words spoken by the examinee.

### Procedure

Participants were screened for cognitive functioning as described above. Participants were designated as having normal cognition (NC) or 1 of 3 different MCI subtypes: aMCI (impairments on 2 or more memory tests but no other cognitive domains); multidomain mild cognitive impairment (2 or more impairments on both memory and other cognitive domains); nonamnestic mild cognitive impairment (NAMCI; impairments on 2 nonmemory cognitive domains, but no more than 1 memory domain). Normative standards for the screening tests were used to evaluate performance, with impairment defined as SD 1 below normative values on each test. The LASSI data for participants with NAMCI were collected, but their data was not examined because the LASSI-L is focused on aMCI. We did not inform the research participants whose data were being excluded because they may have known other participants and discussed the research procedures with them. NC participants were required to have a MoCA score of 26 or more, which was downwardly adjusted due to low levels of education/literacy present in the samples at our community centers.

A fully bilingual testing assistant was present during all assessment sessions and administered all the paper-and-pencil screening tests as well as the LASSI-L. At the end of the first assessment with the LASSI, participants also were administered the ADAS-Cog. Testing order of the LASSI variants was sequentially based, presented in Figure S1 in Multimedia Appendix 1, and is separated by 2 weeks. We started with the LASSI-L and tested until we had at least 50 confirmed eligible participants (MoCA >19, list A2 recognition >10, does not meet criteria for NAMCI). Next, we administered the LASSI-D-form A, until we were certain we had 50 eligible participants. Because of delays in confirmation of MCI status, more than 50 cases were in each of the final samples. We did not attempt to balance the samples for potential factors (language, aMCI vs NC, sex). Baseline performance on forms B and C and weeks 2, 4, and 6 reassessments with the alternative forms of the LASSI-D will be presented in a comprehensive paper on alternative forms and test-retest effects when the study is completed.

### Data Analyses

The goal of the analyses was to examine the similarity of the LASSI-L and LASSI-D, form A (identical stimuli to LASSI-L). Analyses were performed on the 6 critical variables, cued recall of list B trial 1 (B1), cued recall 2 of list B (B2), intrusions on list B trials 1 (B1) and 2 (B2), and percentage of intrusion errors (PIE) for list B trials 1 and 2 (PIE1 and PIE2) for participants who achieved a qualifying cued A2 score. We used *t* tests to compare performance of NC and aMCI participants on all 6 variables in each of the 2 forms (LASSI-L vs LASSI-D form A). We then compared the difference between aMCI and NC for each of the 6 variables within each version. Then we examined the correlations of the 6 variables with other indicators of cognitive impairment (ADAS-Cog and MoCA) separately for the LASSI-L and LASSI-D. To confirm that the samples were comparable in their overall cognitive performance, the NC and aMCI within the were compared for similarity of performance on the MoCA, the TMT-A, and animal naming. Final analyses included examination of classification accuracy, using forward entry stepwise discriminant function analyses to examine the ability of the 6 LASSI variables in each version to classify participants as aMCI versus NC. We then added ADAS-Cog scores to the classification model to see if classification was improved by a standard cognitive assessment targeting AD/ADRD. We used receiver-operating characteristic curve analyses to examine correct classification of aMCI and NC, collapsing across the 2 versions of the LASSI.

### Ethical Considerations

The Western Coppernicus Group Institutional Review Board approved the study (approval number 2022‐1540) and all participants provided signed informed consent. All data were fully anonymized for storage and analysis. Participants received $40.00 in compensation for each assessment session.

## Results

### Participant Characteristics

There were 9 NAMCI participants scheduled for initial testing with the LASSI-L and 4 NAMCI participants scheduled for initial assessment with the LASSI-D. There were also 30 participants who did not meet the list A learning criteria. When the MoCA scores of the 30 participants who did not meet the minimum LASSI learning score were compared with those who did, those who failed to qualify had significantly lower MoCA scores (mean 21.03, SD 2.8), compared with those who qualified (mean 24.21, SD 2.8; *t*_140_=5.45; *P*<.001).

Table 1 presents information on the eligible participants, characterized by which version of the LASSI they received at their assessment. There were no significant differences in age, education, and MoCA scores across the participant samples. There were slightly more Spanish speakers in the LASSI-D cohort, but there were no differences in any of the 6 critical LASSI variables, age, or MoCA scores across the Spanish and non-Spanish speakers (all *t*_111_<1.64; all *P*>.10).

Table 2 presents the results of the comparison of performance across the 2 forms of the LASSI.

**Table 1.** Demographic information on otherwise eligible participants meeting list A learning criteria on Loewenstein-Acevedo Scales for Semantic Interference and Learning (LASSI-L) and digital Loewenstein-Acevedo Scales for Semantic Interference (LASSI-D).

		LASSI-L (n=54)		LASSI-D (n=58)		*t* value (*df*)	*P* value
Age (years), mean (SD)		73.88 (7.09)		72.12 (7.29)		0.65 (111)	.52
MoCA^a^ scores, mean (SD)		23.58 (3.31)		24.38 (3.23)		1.4 (111)	.17
Sex (female), n (%)		44 (82)		45(77)		—^b^	—
Ethnicity (Hispanic), n (%)		16 (29)		44 (75)		—	—
Spanish speakers, n (%)		8 (15)		26 (45)		—	—
Race, n (%)							
	Black	20 (38)		12 (21)		—	—
	White	29 (45)		44 (76)		—	—
	Asian	5 (7)		2 (4)		—	—
Educational attainment, n (%)							
	Less than high school	4 (7)		7 (12)		—	—
	High school	8 (14)		12 (20)		—	—
	Some college	16 (28)		16 (27)		—	—
	College graduate	12 (22)		13 (22)		—	—
	Higher degree	14 (27)		10 (18)		—	—

a MoCA: Montreal Cognitive Assessment.

b Not available.

**Table 2.** Performance of participants tested with the Loewenstein-Acevedo Scales for Semantic Interference and Learning (LASSI-L) compared with the digital Loewenstein-Acevedo Scales for Semantic Interference (LASSI-D).

Performance	LASSI-L				LASSI-D			
	NC^a^ (n=30), mean (SD)		aMCI^b^ (n=24), mean (SD)		NC (n=28), mean (SD)		aMCI (n=30), mean (SD)	
Cued recall list B trial 1	8.57 (2.22)		6.36 (2.3)		7.89 (2.33)		5.66 (2.56)	
Cued recall list B trial 2	12.2 (1.61)		9.61 (1.81)		12.1 (2.22)		9.72 (2.29)	
Intrusions cued recall list B trial 1	2.67 (2.14)		4.36 (3.47)		2.18 (1.54)		3.5 (2.69)	
Intrusions cued recall list B trial 2	2.03 (1.79)		2.88 (2.51)		1.5 (1.93)		2.78 (2.29)	
PIE^c^ list B trial 1	0.23 (0.18)		0.38 (0.22)		0.22 (0.16)		0.37 (0.23)	
PIE list B trial 2	0.14 (0.11)		0.25 (0.15)		0.13 (0.14)		0.25 (0.17)	

a NC: normal condition.

b aMCI: amnestic mild cognitive impairment.

c PIE: percentage of intrusion errors in relation to total correct responses on the trial.

### Performance Differences Across Test Forms

The largest difference between the LASSI-L and LASSI-D on any variable was cued recall B1 for the MCI group, with an effect size of *d*=0.3 (*t*_56_=1.13; *P*=.13), with better performance on the LASSI-L. All of the other 5 *t* tests were also nonsignificant (*P*>.05). Scores on the 2 critical variables of percentage intrusion errors were essentially identical across both versions of the LASSI for both participant groups (*P*>.71).

Table 3 presents the results of comparisons of form differences across NC and MCI participants for all the critical variables as well as diagnostic differences for each of these variables.

**Table 3.** Effect sizes for form differences and diagnostic status differences. Effects sizes are presented as Cohen *d.*

LASSI^a^ variables	Form differences		Diagnosis differences	
	NC^b^ (n=58), Cohen *d*	MCI^c^ (n=54), Cohen *d*	NC (n=54), Cohen *d*	MCI (n=58), Cohen *d*
Cued recall list B trial 1	0.29	0.3	0.97	0.91
Cued recall list B trial 2	0.06	0.05	1	1.01
Intrusions cued recall list B trial 1	0.25	0.25	0.97	0.91
Intrusions cued recall list B trial 2	0.25	0.04	0.7	0.68
PIE^d^ list B trial 1	0.06	0.04	0.71	0.75
PIE list B trial 2	0.1	0.02	0.83	0.81

a LASSI: Loewenstein-Acevedo Scales for Semantic Interference

b NC: normal condition.

c MCI: mild cognitive impairment.

d PIE: percentage of intrusion errors in relation to total correct responses on the trial.

Performance of the aMCI participants was worse than the NC participants on all LASSI variables with the 2 forms combined. The smallest difference between aMCI and NC participants across the forms was for intrusions on cued recall B2, which had an effect size of *d*=0.68 (*t*_56_=2.3; *P*=.03). All other tests had larger effect sizes, higher *t* statistics, and lower *P* values.

As can be seen in Table 4, every LASSI-L and LASSI-D variable was significantly correlated with MoCA and ADAS-Cog scores in the combined sample. Correlations were smaller for the intrusion variables than for the learning variables comprised of correct responses.

**Table 4.** Pearson correlations with other cognitive indicators collapsed across diagnosis and separated by which test was administered first.

LASSI^a^ Variables	MoCA^b^		ADAS-Cog^c^	
	LASSI-L^d^ (n=54), Pearson *r*	LASSI-D^e^ (n=58), Pearson *r*	LASSI-L (n=54), Pearson *r*	LASSI-D (n=58), Pearson *r*
Cued recall list B trial 1	0.62^f^	0.53^f^	−0.62^f^	−0.54^f^
Cued recall list B trial 2	0.61^f^	0.54^f^	−0.63^f^	−0.53^f^
Intrusions cued recall list B trial 1	−0.29^g^	−0.30^g^	0.38^g^	0.26^g^
Intrusions cued recall list B trial 2	−0.29^g^	−0.31^g^	0.30^g^	0.24^g^
PIE^h^ list B trial 1	−0.43^f^	−0.34^g^	0.47^f^	0.38^g^
PIE list B trial 2	−0.33^g^	−0.41^f^	0.42^f^	0.31^g^

a LASSI: Loewenstein-Acevedo Scales for Semantic Interference.

b MoCA: Montreal Cognitive Assessment.

c ADAS-Cog: Alzheimer’s Disease Assessment Scale-Cognitive Subscale.

d LASSI-L: Loewenstein-Acevedo Scales for Semantic Interference and Learning.

e LASSI-D: digital Loewenstein-Acevedo Scales for Semantic Interference.

f *P*<.001.

g *P*<.01.

h PIE: percentage of intrusion errors in relation to total correct responses on the trial.

We performed comparative analyses of the 2 samples across the cognitive variables administered at each assessment and at baseline and present them in Table S1 in Multimedia Appendix 2. As can be seen in that table, there were no significant differences in any of the variables across the 2 samples of participants.

**Figure 2.**
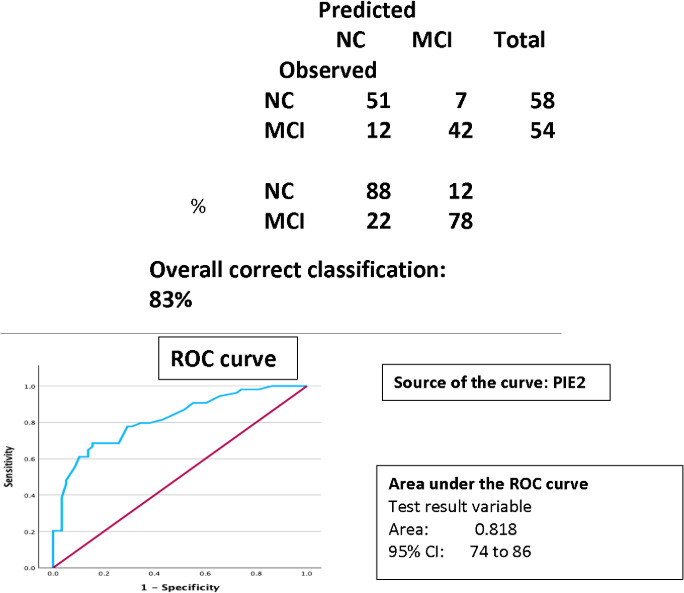
Classification accuracy for amnestic mild cognitive impairment versus normal control status including area under the curve. MCI: mild cognitive impairment; NC: normal condition; PIE: percentage of intrusion errors in relation to total correct responses on the trial; ROC: receiver operating characteristic.

Finally, Figure 2 presents the results of discriminant analyses using the PIE intrusion variables to classify cases into aMCI versus NC status. The PIE2 intrusion variable was the only one to enter the analysis (Wilks Lambda=7.03; *χ*^2^_1_=38.6; Pillas approx. *F*_1,110_=46.49; *P*<.001). Correct classification of the cases was 88% (51/58) for NC (7/58, 12% false positive) and 78% (42/54) for MCI (12/54, 22% false negative) for total correct classification of cases of 83% (93/112). Adding the ADAS-Cog as an additional predictor did not change the results, as it did not enter the equation (*F*_1,110_ =3.85; *P*=.06). The area under the receiver operating characteristic curve (AUC) statistics are also presented in Figure 2. Importantly, the AUC statistic overall was 0.82, consistent with the overall correct classification rate.

When the samples were split into subgroups based on LASSI-L or LASSI-D first and the analyses rerun, only PIE2 intrusion errors again entered the analysis at *P*<.001 in both subgroups. Classification accuracy was 77% (23/30) for NC and 66% (16/24) for aMCI in the LASSI-L first group and 82%(23/28) for NC and 77% (23/30) for aMCI. Despite the smaller size of these samples, the data suggest quite similar classification accuracy for each version of the LASSI.

## Discussion

This study examined convergence between a legacy paper-and-pencil assessment sensitive to the earliest stages of aMCI and decline over time and a digital remotely deliverable version of the assessment. Our goal was to determine if the scores obtained across legacy and migrated measures suggest that they are essentially equivalent alternative forms of the same test. The results suggested a high level of similarity of performance on the legacy and migrated versions of the LASSI across all critical variables. There were no significant differences between paper and digital forms for any variable within cognitive status subgroups. Furthermore, all variables manifested a moderate to large effect size for the performance differences between the aMCI and NC participants.

The variables that optimally classified the groups, were the same as in the previous studies of the LASSI-L, the percentage of intrusion errors on the second recall trial of list B. Interestingly, adding the gold standard ADAS-Cog to the models did not improve classification accuracy based on the PIE2 intrusion variable. The remarkable similarity of the PIE1 and PIE2 intrusion scores across legacy and migrated versions of the LASSI in this study compared with previous studies merits comment. In the Crocco et al [16] study, longitudinally stable cognitively normal participants received PIE1 scores of 0.22 and PIE2 scores of 0.11 when tested with the paper-and-pencil LASSI-L and participants who later declined into aMCI received baseline PIE1 scores of 0.38 and PIE2 scores of 0.22. These scores are essentially identical to LASSI-L scores in the current study for cognitively normal participants (PIE1=0.22 and PIE2=0.11) and for aMCI participants (PIE1=0.38 and PIE2=0.25). The LASSI-D scores are also equivalent to the current and previous LASSI-L scores (NC: PIE1=0.22, PIE =0.13; aMCI PIE1=0.37; PIE2=0.25), suggesting similarity to previous and current results.

Classification accuracy into aMCI and NC groups with discriminant analysis based on LASSI criteria alone was similar, although somewhat reduced compared with previous studies. For instance, in the Crocco et al [16] study, sensitivity was 89% for NC and specificity was 86%, while in the current study detection accuracy for NC was essentially identical at 88% (51/58) and lower for identification of aMCI at 78% (42/54). This difference likely lies in the definition of aMCI. In previous studies, such as Crocco et al, ascertainment of cognitive status included the full recommended strategy of the National Alzheimer’s Coordinating Center dataset [37], which includes assessment of subjective cognitive decline, a neurological assessment, and a reliable collateral informant resulting in a Clinical Dementia Rating score [38], as well as a neuropsychological assessment. Although our study identified NC with essentially identical accuracy compared with previous studies, some individuals with aMCI were not identified, likely due to differences in ascertainment criteria.

Our study was performed at nonclinical community centers. In this regard, it is an ecologically valid examination of the LASSI-D as the administration protocol is like the assessments available in these centers, where there are no professional assessors. In fact, the rate of false positive diagnosis of NC as aMCI is the same as in an ADRC-quality sample. When balancing the risk of false negative assessments versus likely impossibility of ADRC-quality assessments in community centers, a reduced detection accuracy of 10% appears to be a costeffective trade-off.

We are currently evaluating alternative forms of the LASSI-D, for repeated administration. Such repeated assessments will be required to screen and monitor cognitive change as the field moves forward with developing therapeutics for AD/ADRD. We are also collecting blood biomarkers (p-tau 217) on participants to examine convergence of the LASSI-D results with state-of-the-art AD biomarkers. Spanish and English versions of the LASSI generated similar scores, although analyses of task × MCI status will need to be repeated across the other forms. In summary, these data suggest that scores obtained from professional tester administered, manually scored LASSI-L assessments to the self-administered LASSI-D have excellent convergence and discriminant validity.

## Supplementary material

10.2196/64716 Multimedia Appendix 1 Protocol assessment sequence for digital migration.

10.2196/64716 Multimedia Appendix 2 Sample similarity on other cognitive outcomes for participants who received the LASSI-L first or the LASSI-D first.

## Abbreviations

AD: Alzheimer disease
ADAS-Cog: Alzheimer Disease Assessment Scale- Cognition
ADRD: Alzheimer disease–related disorders
aMCI: amnestic mild cognitive impairment
BAC: Brief Assessment of Cognition
frPSI: failure to recover from proactive semantic interference
LASSI: Loewenstein-Acevedo Scales for Semantic Interference
LASSI-D: digital Loewenstein-Acevedo Scales for Semantic Interference
LASSI-L: Loewenstein-Acevedo Scales for Semantic Interference and Learning
MCI: mild cognitive impairment
MoCA: Montreal Cognitive Assessment
NAMCI: nonamnestic mild cognitive impairment
NC: normal condition
PIE: percentage of intrusion errors
PSI: proactive semantic interference
