# Supplementary material for: Digital Migration of the Loewenstein Acevedo Scales for Semantic Interference and Learning (LASSI-L): Development and Validation Study in Older Participants
Source: JMIR Ment Health. 2025 Feb 19;12:e64716. doi: 10.2196/64716 (PMC11864698; doi:10.2196/64716)
Supplement: Multimedia Appendix 1 [file mental-v12-e64716-s001.pdf]

Supplemental Figure 1.

Protocol Assessment Sequence for Digital Migration

Block 1
First Assessment
LASSI-L
Second Assessment: Counterbalanced Across Participants
LASSI-D form A (33%)
LASSI-D form B (33%)
LASSI-D form C (33%)

Block 2
First Assessment
LASSI-D Form A
Second Assessment: Counterbalanced Across Participants
LASSI-L (33%)
LASSI-D form B (33%)
LASSI-D form C (33%)

Note. Blocks 3 and 4 were similarly allocated.
