# Supplementary material for: Digital Migration of the Loewenstein Acevedo Scales for Semantic Interference and Learning (LASSI-L): Development and Validation Study in Older Participants
Source: JMIR Ment Health. 2025 Feb 19;12:e64716. doi: 10.2196/64716 (PMC11864698; doi:10.2196/64716)
Supplement: Multimedia Appendix 2 [file mental-v12-e64716-s002.pdf]

Supplemental Table 1

Sample Similarity on Other Cognitive Outcomes for Participants who Received the LASSI-L™ First or the LASSI-D™ First

	LASSI-L™ First n=54		LASSI-D™ First n=58			
	M	SD	M	SD	t	p
Trail Making Test Part A	48.26	23.89	49.07	35.44	0.15	.88
Animal Naming	18.84	6.47	19.68	6.03	0.80	.43
MoCA Score	23.61	3.45	23.58	3.41	0.06	.96
ADAS-Cog Total Score	7.16	3.26	7.41	3.50	0.12	.91
